# Decreased systemic bioavailability of L-arginine in patients with cystic fibrosis

**DOI:** 10.1186/1465-9921-7-87

**Published:** 2006-06-09

**Authors:** Hartmut Grasemann, Raphael Schwiertz, Corinna Grasemann, Udo Vester, Kurt Racké, Felix Ratjen

**Affiliations:** 1 Children's Hospital, University of Duisburg-Essen, Essen, Germany; 2 The Hospital for Sick Children, University of Toronto, Toronto, ON, Canada; 3 Institute for Pharmacology and Toxicology, University of Bonn, Germany

## Abstract

**Background:**

L-arginine is the common substrate for nitric oxide synthases and arginases. Increased arginase levels in the blood of patients with cystic fibrosis may result in L-arginine deficiency and thereby contribute to low airway nitric oxide formation and impaired pulmonary function.

**Methods:**

Plasma amino acid and arginase levels were studied in ten patients with cystic fibrosis before and after 14 days of antibiotic treatment for pulmonary exacerbation. Patients were compared to ten healthy non-smoking controls.

**Results:**

Systemic arginase levels measured by ELISA were significantly increased in cystic fibrosis with exacerbation compared to controls (17.3 ± 12.0 vs. 4.3 ± 3.4 ng/ml, p < 0.02). Arginase levels normalized with antibiotic treatment. Plasma L-arginine was significantly reduced before (p < 0.05) but not after treatment. In contrast, L-ornithine, proline, and glutamic acid, all downstream products of arginase activity, were normal before, but significantly increased after antibiotic therapy. Bioavailability of L-arginine was significantly reduced in cystic fibrosis before and after exacerbation (p < 0.05, respectively).

**Conclusion:**

These observations provide further evidence for a disturbed balance between the L-arginine metabolic pathways in cystic fibrosis.

## Background

Nitric oxide (NO) is a messenger molecule that is involved in a variety of biological and physiological processes in the lung. Constitutive endogenous formation of NO in airways is thought to play a role in neurotransmission, smooth muscle relaxation and bronchodilation. [1] Airway NO formation by NO synthases (NOSs) can be increased in response to inflammatory mediators predominantly through induction of the calcium-independent isoform NOS2. [1] Despite the inflammatory nature of lung disease in cystic fibrosis (CF), fractional exhaled NO (FE_NO_), as well as concentrations of the bioactive NO-metabolites S-nitrosothiols (SNOs) in airway fluids are decreased in CF patients. [2-4] While the reasons for impaired NO formation remain incompletely understood, there is accumulating evidence that low airway NO contributes to lung pathophysiology in CF. [5-9]

Enzymatic NO formation from NOSs requires the semi-essential amino acid L-arginine as substrate. L-arginine is also metabolized by an enzyme of the urea cycle named arginase which exists in type I and type II isoforms. While arginase II is located mitochondrial, arginase I is a cytoplasmatic enzyme. Arginase can limit NO formation through increased consumption of the common substrate. [10-12] It was recently shown that arginase activity is increased in sputum of patients with CF. [9] Evidence for increased systemic arginase activity in CF came from a study showing that oral supplementation of L-arginine in a single dose of 200 mg/kg body weight resulted in a significantly higher increase of L-ornithine, the product of arginase activity, in plasma of CF patients compared to healthy controls. [13] These data suggest that the consumption of L-arginine by arginase could be increased in CF, possibly resulting in reduced availability of L-arginine for NO synthesis. To address this question, we measured plasma amino acid levels and arginase concentrations in patients with CF before and after antibiotic treatment for pulmonary exacerbations.

## Methods

### Study cohort and protocol

10 CF patients (3 females) aged 16 to 39 years with a mean (± SD) age of 21.9 (± 6.2) years were included in this study. The diagnosis of CF had been confirmed in all patients by repeated sweat tests with chloride concentrations exceeding 60 mmol/L and by CFTR gene mutation analysis. Exclusion criteria were g-tube feeding, allergic bronchopulmonary aspergillosis or *B. cepacia *infection. Patients studied were admitted to hospital for intravenous antibiotic treatment of a pulmonary exacerbation. Blood was drawn into two 2.7 ml EDTA containing tubes for plasma amino acid (n = 10) and arginase (n = 7) measurements on the morning after admission and after 14 days of antibiotic treatment under fasting conditions in the morning prior to breakfast, respectively. Pulmonary function tests by spirometry were performed on the same day in each patient but one, who was unable to perform spirometry. Mean forced vital capacity (FVC) at admission was 54.1 (± 22.7) % and FEV_1 _was 32.4 (± 10.6) % of predicted values, respectively. Mean BMI of the patients was 18.5 (± 2.2). Sputum microbiology revealed *P. aeruginosa *in 8 patients, *Acinetobacter baumanii *in 1 and *Stenotrophomonas *plus *Staph. aureus *in one patient. Patients were treated with ceftazidime (n = 5) or meropenem (n = 5) plus an aminoglycoside respectively. One patient received oral steroids on three days during treatment, two additional patients were on long term treatment with inhaled budesonide.

Patients were compared to 10 (3 females) non-smoking, healthy controls that were 23 to 28 years (24.4 ± 2.1 years) of age. In controls blood was also drawn into two 2.7 ml EDTA containing tubes in the morning under fasting conditions. Written informed consent was obtained from each CF patient, and/or their parents, and all healthy controls, respectively. The study was approved by the institutional review board of the University of Duisburg-Essen.

### Plasma amino acids measurements

Amino acids were determined by ion exchange chromatography on an amino acid analyzer LC 3000 (Eppendorf, Hamburg, Germany) according to the manufacturer's specifications. Plasma samples were deproteinized within 30 minutes after collection.

### Arginase ELISA

Plasma levels of arginase I (liver type) were measured using a commercially available enzyme-linked immonosorbent assay (ELISA) kit (BioVendor Laboratory Medicine, Inc. Brno, Czech Republic). Plasma samples for the arginase ELISA were centrifuged at 1000 g for 10 min immediately after they were obtained. Clear supernatant was kept at -80C before analyzed.

### Statistics

Data were expressed as mean ± standard deviation (SD). Kolmogorov-Smirnov test revealed normal distribution of all values in the groups. Comparisons within groups (pre vs. post treatment) were therefore made by paired t-test and inter-group comparisons were done by unpaired t-test. A p value below 0.05 was considered statistically significant. PC-Statistik v2.11 (TopSoft Hannover, Germany) was used for statistical analysis.

## Results

### Amino acid levels

**Figure 1:**
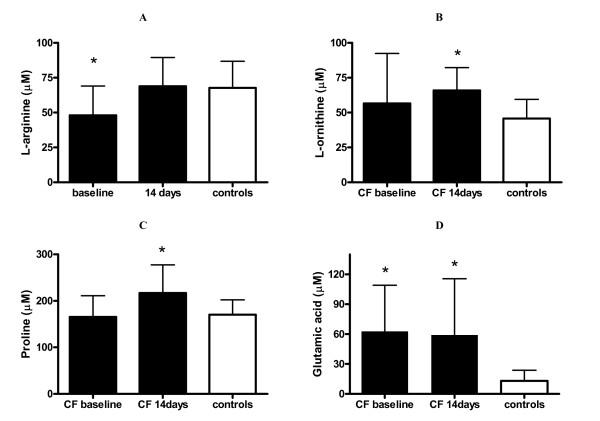
**Plasma amino acids**. Plasma concentrations of L-arginine (A), L-ornithine (B), proline (C), and glutamic acid (D) in cystic fibrosis patients (CF) before (baseline) and after 14 days of antibiotic treatment as well as controls. Results are plotted as means ± SD. Asterisk indicates significant (p < 0.05) difference to controls.

Significantly reduced plasma concentrations were found for 9 out of 20 amino acids tested in CF patients admitted with pulmonary exacerbations. With the exception of histidine none of the amino acid levels were subnormal after 14 days of antibiotic therapy. Glycine as well as the downstream products of arginase activity, i.e. L-ornithine, proline, and glutamic acid were not reduced at admission but were significantly increased after antibiotic therapy when compared to controls, respectively (Table 1) (Figure 1).

**Table 1:** Plasma amino acid levels (μM) in controls and CF patients before and after 14 days of i.v. antibiotic treatment for pulmonary exacerbation

		CF patients (n = 10)		
		
	Controls (n = 10)	before (% control)	after (% control)	P-value
L-arginine	67.7 ± 19.0	48.1 ± 19.9 (71) *	68.9 ± 19.5 (102)	0.02
L-ornithine	45.7 ± 13.8	56.6 ± 34.0 (124)	65.9 ± 15.5 (144)†	NS
L-citrulline	27.5 ± 8.8	28.3 ± 5.5 (103)	33.4 ± 5.8 (121)	NS
Threonine	128.1 ± 40.0	87.2 ± 30.7 (68) *	129.7 ± 50.4 (101)	0.01
Serine	97.7 ± 30.0	92.0 ± 26.3 (94)	92.9 ± 17.2 (95)	NS
Asparagine	52.5 ± 14.5	37.3 ± 12.4 (71) *	40.0 ± 11.0 (76)	NS
Glutamic Acid	18.6 ± 7.1	69.7 ± 40.7 (375) †	65.7 ± 52.5 (353)*	NS
Proline	170.3 ± 31.8	165.4 ± 43.2 (97)	216.8 ± 57.5 (127)*	NS
Glycine	205.1 ± 52.9	241.8 ± 100.6 (118)	323.5 ± 69.1 (158)†	0.02
Alanine	328.5 ± 73.6	243.1 ± 90.8 (74) *	355.3 ± 139.6 (108)	NS
Valine	203.0 ± 25.0	169.7 ± 32.1 (84) *	176.1 ± 39.6 (87)	NS
Cysteine	55.3 ± 15.6	30.1 ± 9.0 (54) †	42.4 ± 13.7 (77)	0.004
Methionine	24.9 ± 4.8	19.2 ± 5.2 (77) *	21.0 ± 5.6 (84)	NS
Isoleucine	57.6 ± 10.9	52.3 ± 18.1 (91)	62.9 ± 12.9 (109)	NS
Leucine	111.3 ± 22.2	90.4 ± 25.1 (81)	96.1 ± 23.9 (86)	NS
Tyrosine	54.7 ± 9.8	45.7 ± 14.3 (84)	44.7 ± 11.3 (82)	NS
Phenylalanine	49.9 ± 6.8	47.7 ± 10.7 (96)	49.0 ± 10.3 (98)	NS
Histidine	83.7 ± 7.2	55.9 ± 17.7 (67) †	64.4 ± 17.6 (77)†	NS
Tryptophan	49.8 ± 9.5	30.7 ± 15.8 (62) †	46.6 ± 11.7 (94)	0.02
Lysine	144.7 ± 42.5	144.0 ± 40.5 (100)	183.8 ± 51.9 (127)	NS

Concentrations of amino acids are expressed in means ± SD. Symbols indicate significant differences of CF group to controls; *: p < 0.05, †: p ≤ 0.01. P-values shown are for comparison between CF groups before and after antibiotic treatment.

**Figure 2:**
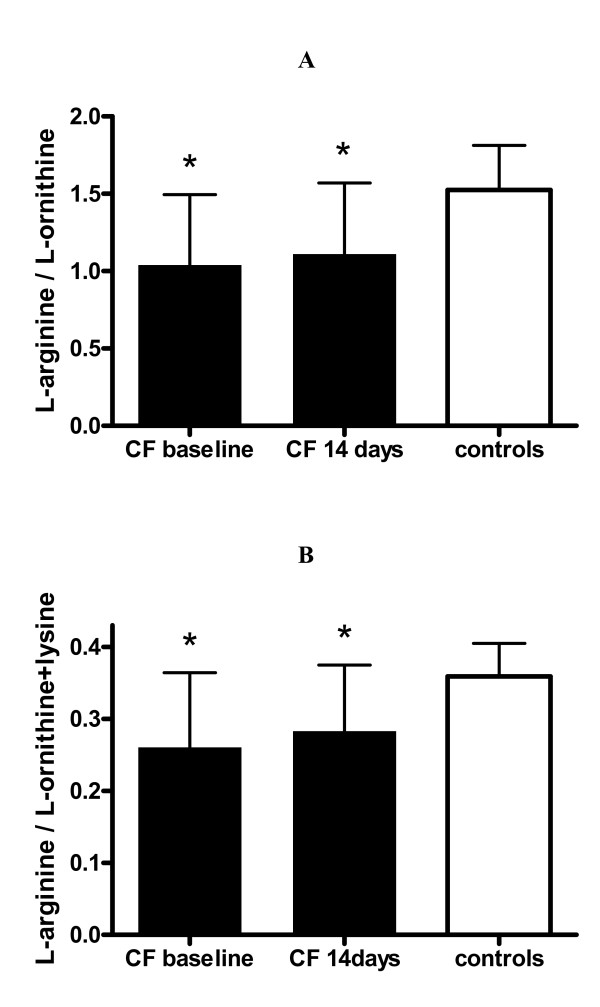
**L-arginine bioavailability**. L-arginine bioavailability indices in CF patients before (baseline) and after 14 days of antibiotic treatment and in controls. Results are plotted as means ± SD. Asterisk indicates significant (p < 0.05) difference to controls.

L-arginine plasma concentrations were decreased in CF patients with an exacerbation when compared to controls, with three patients having values of 13.6 μM, 21.2 μM, and 35.1 μM respectively, which is below published reference values (36–139 μM). [14] Plasma L-arginine significantly increased to normal values in CF during antibiotic therapy (Figure 1). The rate limiting factor for cellular uptake of L-arginine is the CAT transporter, which also transports L-ornithine and lysine. [15] The ratios of L-arginine/L-ornithine, as well L-arginine/L-ornithine + lysine, therefore, provide indices of relative L-arginine availability at any given plasma L-arginine concentration. Both these ratios were significantly reduced in the CF patients before and after treatment for pulmonary exacerbation (Figure 2).

### Arginase concentrations

**Figure 3:**
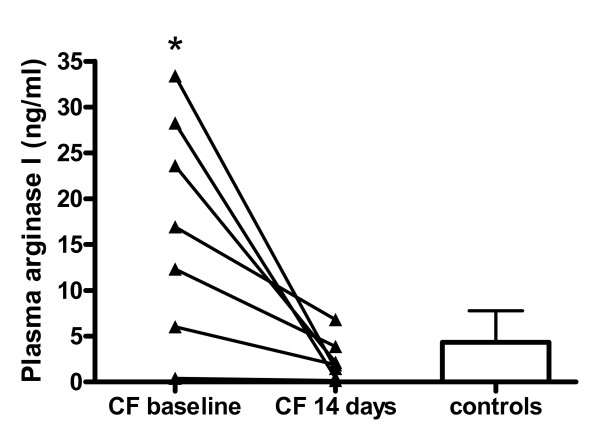
**Arginase I in plasma**. Arginase I plasma concentrations measured by ELISA in CF patients before (baseline) and after 14 days of antibiotic treatment and in controls. Results are plotted as means ± SD. Asterisk indicates significant (p < 0.05) difference to controls.

Plasma arginase concentrations in healthy controls were 4.3 ± 3.4 ng/ml. Arginase levels were significantly increased in CF patients with pulmonary exacerbation (17.3 ± 12.0 ng/ml, p < 0.02) and returned to normal values after 14 days of antibiotic therapy (2.4 ± 2.3 ng/ml) (Figure 3).

## Discussion

In this study we found evidence for reduced systemic availability of L-arginine, the substrate for NOS, in CF patients. While increased arginase I and decreased L-arginine concentrations in plasma at the time of pulmonary exacerbation normalized with antibiotic treatment, the relative bioavailability of L-arginine remained significantly reduced in CF patients. This was associated with an amino acid profile consistent with increased arginase activity even after treatment for pulmonary exacerbation.

L-arginine is a semi-essential amino acid that is found in many comestible goods but can also be biosynthesized from citrulline (by argininosuccinate synthase and argininosuccinate lyase, the third and fourth enzymes of the urea cycle). L-arginine is essential for protein synthesis and a substrate for urea, polyamines, proline, L-glutamine, creatinine phospate, agmatine as well as enzymatic NO formation. Arginine is transported from blood into cells by cationic amino acid transporter (CAT) isoforms. The availability of L-arginine is one of the rate-limiting factors in cellular NO production. [10,12] Therefore, L-arginine deficiency may, at least in part, account for the reduced NO formation seen in the CF airways.

A lack of NO formation is of relevance for CF pathophysiology since there is increasing evidence that decreased NO formation has a negative effect on defense against bacterial infections, and contributes to airway obstruction in CF. [2,5,7,9,16-18] However, most actions of NOSs seem to be mediated by S-nitrosylated proteins and not by NO itself [19]. Depletion of S-nitrosothiols (SNOs) is thought to aggravate airway obstruction [20-22], and SNO levels were found to be decreased in CF airways [3]. Furthermore, SNOs have been shown to increase expression, maturation and function of both wild-type and Δ F508 CFTR. [23-25] Of interest, cysteine, which contains a thiol group, is the rate limiting amino acid in the synthesis of glutathione (GSH), [26] an antioxidant which contributes to the redox imbalance in CF. The low cysteine plasma concentrations seen in the CF patients studied here, may therefore not only contribute to low SNOs but also reduced GSH formation and consequently GSNO deficiency in CF.

Low L-arginine concentrations may, however, not only result in decreased NO or SNO formation but also in increased reactive oxygen nitrogen species that contribute to tissue damage. Although superoxide generation from NOS3 appears to be regulated by BH_4 _rather than L-arginine, [27] it is known that calmodulin-bound NOS can release reactive oxygen species such as superoxide and H_2_O_2 _at non-saturating L-arginine levels, [28,29] and that NOS can generate NO and superoxide at the same time at low-saturating L-arginine levels. [30,31] These products may react to generate peroxynitrite, and subsequently result in tyrosine nitration. In fact, nitrotyrosine has been found to be increased in CF lung tissue, [32] sputum, [33] and exhaled breath condensate from CF patients. [34]

Our data suggest that L-arginine deficiency in CF patients results from increased L-arginine consumption by arginase. The product of arginase activity, L-ornithine, is a precursor for the formation of polyamines (e.g., putrescine, spermidine, and spermine) and proline, which control cell proliferation and collagen production, respectively. [35] Polyamines may be particularly important in the lung because respiratory epithelial cells have a potent polyamine uptake system. [36] In addition, the polyamine spermine has been shown to down-regulate NO synthesis and cellular L-arginine transport by suppressing the expression of NOS2 and CAT-2B in alveolar macrophages. [37] Of interest, polyamine plasma levels have been reported to be increased in CF in an age-dependent manner. [38]

This is the first study to show that arginase I concentrations are increased in blood of CF patients with pulmonary exacerbation. Increased circulating arginase levels may explain why mean plasma L-arginine concentrations were decreased at this time point while arginase down-stream products were either normal (L-ornithine and proline) or increased (glutamic acid). It could also be argued that the initial decrease of several non-related amino acids followed by normalization during therapy may reflect an unspecific effect such as catabolic state related to infection or inflammation. However, the increase of L-ornithine and proline after antibiotic therapy in the face of normalized plasma L-arginine and arginase I levels provide evidence that arginase activity persists in compartments of these patients which are not reflected in measuring arginase in plasma. Evidence for increased arginase activity in clinically stable CF patients came also from a previous study showing a higher increase in plasma L-ornithine after a single dose of oral L-arginine in CF patients compared to healthy controls. [13]

Arginase I is usually located intracellular with high concentrations in erythrocytes and liver cells. We could recently demonstrate that the activity of arginase was high in CF sputum and, although decreasing with antibiotic treatment, remained significantly increased (approx. 10-fold) when compared to induced sputum from healthy controls. [9] Thus, increased plasma concentrations of arginase down-stream products (L-ornithine, proline, and glutamic acid) in the present of normal plasma arginase I levels could be explained by a "spill-over effect" of these metabolites from the CF airways into the blood stream. This mechanism may also explain the origin of increased plasma arginase I levels in the patients. Other sources of arginase activity could be tissues expressing arginase II or bacteria such as *P. aeruginosa*, but these were not detectable by the human arginase I ELISA used in this study.

Increased systemic arginase activity and decreased L-arginine bioavailability has recently also been found to be involved in the pathophysiology of sickle cell disease and asthma. [39,40]. In sickle cell disease hemolysis-related release of erythrocyte arginase was significantly correlated with severity of pulmonary hypertension and increased mortality. [40] Animal experiments in a rodent model of allergic asthma suggest that increased arginase results in reduced L-arginine availability to constitutive NOS and thereby contributes to airway obstruction. [41,42] In line with these experiment, increased arginase and decreased L-arginine bioavailability has been found in asthma patients. [39,43] These observations suggest that L-arginine deficiency from increased arginase activity is not unique to CF but may play an important role in the pathogenesis of disease conditions with increased smooth muscle contractility.

## Conclusion

These data provide evidence that the availability of L-arginine is reduced in patients with CF. In addition, high arginase concentrations in the blood in CF patients with pulmonary exacerbation results in systemic L-arginine deficiency and may thereby contribute to CF pathophysiology.

## Competing interests

The author(s) declare that they have no competing interests.

## Authors' contributions

HG conceived of the study, participated in the design of the study and drafted the manuscript. RS coordinated the study, handled and prepared the patient samples for the analyses. CG carried out the arginase measurements in plasma. UV participated in the plasma amino acid analyses and data interpretation. KR contributed to the design of the study and critically revised the manuscript. FR participated in designing the study and helped to draft the manuscript. All authors read and approved the final version of the manuscript.
